# Long Term Mortality in Critically Ill Burn Survivors

**DOI:** 10.1186/2197-425X-3-S1-A32

**Published:** 2015-10-01

**Authors:** SL Nitzschke, AC Offodile II, FK Gibbons, A Salim, KB Christopher

**Affiliations:** Brigham and Women's Hospital, Division of Trauma, Burn, and Surgical Critical Care, Boston, United States; Lahey Hospital and Medical Center, Department of Plastic and Reconstructive Surgery, Burlington, United States; Massachusetts General Hospital, Pulmonary and Critical Care Medicine, Boston, United States; Brigham and Women's Hospital, Renal Division, Boston, United States

## Introduction

Little is known about long term survival risk factors in critically ill burn patients who survive hospitalization.

## Objectives

We hypothesized that patients with major burns who survive hospitalization would have favorable long term outcomes.

## Methods

We performed a two center observational cohort study between 1998-2007 in 365 critically ill adult burn patients who survived to hospital discharge. The exposure of interest was major burn defined a priori as > 20% total body surface area burned [TBSA]. The modified Baux score was determined by age + %TBSA+ 17(inhalational injury). The primary outcome was all-cause 5 year mortality based on the US Social Security Administration Death Master File. Adjusted associations were estimated through fitting of multivariable logistic regression models. Time-to-event analysis was performed using Cox proportional hazard regression.

## Results

Of the cohort patients studied, 76% were male, 29% were non white, 14% were over 65, 32% had TBSA>20%, and 45% had inhalational injury. The mean age was 45, 92% had 2nd degree burns, 60% had 3rd degree burns, 21% received vasopressors, and 26% had sepsis. The mean TBSA was 20.1%. The mean modified Baux score was 72.8. Post hospital discharge 5 year mortality rate was 9.0%. The 30 day hospital readmission rate was 4%. Patients with major burns were significantly younger (41 vs 47 years) had a significantly higher modified Baux score (89 vs 62), and had significantly higher comorbidity, acute organ failure, inhalational injury and sepsis (all P < 0.05). There were no differences in gender and the Acute Organ Failure score between major and non-major burns. in a logistic regression model adjusted for inhalational injury, presence of 3^rd^ degree burn, gender and the Acute Organ Failure score, a validated ICU risk-prediction score derived from age, race, surgery vs. medical patient type, comorbidity, sepsis and acute organ failure covariates, major burn was associated with a 3 fold decreased odds of 5 year post-discharge mortality compared to patients with TBSA < 20% [OR = 0.29 (95%CI 0.11-0.78; P = 0.014)]. The adjusted model showed good discrimination [AUC 0.81 (95%CI 0.74-0.89)] and calibration (Hosmer-Lemeshow χ^2^ P = 0.67). Cox proportional hazard multivariable regression modeling, adjusting for inhalational injury, presence of 3^rd^ degree burn, gender and the Acute Organ Failure score, showed that major burn was predictive of mortality following hospital admission [HR = 0.34 (95% CI 0.15-0.76; P = 0.009)]. The modified Baux score was not predictive for mortality following hospital discharge [OR 5 year post-discharge mortality = 1.00 (95%CI 0.99-1.02 ;P = 0.74); HR for post-discharge mortality = 1.00 (95% CI 0.99-1.02; P = 0.55)].

## Conclusions

Critically ill patients with major burns who survive to hospital discharge have decreased 5 year mortality compared to those with less severe burns. The modified Baux score was not predictive for long term outcomes following discharge.

